# Integrative proteomic and phosphoproteomic analysis in the female goat hypothalamus to study the onset of puberty

**DOI:** 10.1186/s12864-023-09705-7

**Published:** 2023-10-18

**Authors:** Jing Ye, Xu Yan, Wei Zhang, Juntai Lu, Shuangshuang Xu, Xiaoqian Li, Ping Qin, Xinbao Gong, Ya Liu, Yinghui Ling, Yunsheng Li, Yunhai Zhang, Fugui Fang

**Affiliations:** 1 Department of Animal Veterinary Science, College of Animal Science and Technology, Anhui Agricultural University, 130 Changjiang West Road, 230036 Hefei, Anhui China; 2 Anhui Province Key Laboratory of Local Livestock and Poultry, Genetical Resource Conservation and Breeding, College of Animal Science and Technology, Anhui Agricultural University, 230036 Hefei, Anhui China

**Keywords:** Hypothalamus, Proteomics, Phosphoproteomics, Puberty

## Abstract

**Background:**

Puberty marks the end of childhood and achieve sexual maturation and fertility. The role of hypothalamic proteins in regulating puberty onset is unclear. We performed a comprehensive differential proteomics and phosphoproteomics analysis in prepubertal and pubertal goats to determine the roles of hypothalamic proteins and phosphoproteins during the onset of puberty.

**Results:**

We used peptide and posttranslational modifications peptide quantification and statistical analyses, and identified 69 differentially expressed proteins from 5,057 proteins and 576 differentially expressed phosphopeptides from 1574 phosphorylated proteins. Combined proteomic and phosphoproteomics, 759 correlated proteins were identified, of which 5 were differentially expressed only at the protein level, and 201 were only differentially expressed at the phosphoprotein level. Pathway enrichment analyses revealed that the majority of correlated proteins were associated with glycolysis/gluconeogenesis, Fc gamma R-mediated phagocytosis, focal adhesion, GABAergic synapse, and Rap1 signaling pathway. These pathways are related to cell proliferation, neurocyte migration, and promoting the release of gonadotropin-releasing hormone in the hypothalamus. CTNNB1 occupied important locations in the protein-protein interaction network and is involved in focal adhesion.

**Conclusion:**

The results demonstrate that the proteins differentially expression only at the protein level or only differentially expressed at the phosphoprotein level and their related signalling pathways are crucial in regulating puberty in goats. These differentially expressed proteins and phosphorylated proteins may constitute the proteomic backgrounds between the two different stages.

**Supplementary Information:**

The online version contains supplementary material available at 10.1186/s12864-023-09705-7.

## Background

Puberty is a transitional period between childhood and adulthood, characterized by the development of secondary sexual characteristics, gonadal maturation, and attainment of reproductive capacity [1]. Unlike the developmental dysplasia caused by precocious puberty in humans, early onset of puberty in livestock can reduce the age at the first litter and prolong the reproductive life to a certain extent [3]. Puberty and reproduction are controlled by the hypothalamic-pituitary-gonadal axis (HPGA) [4]. From a neuroendocrine perspective, the onset of puberty requires activation of hypothalamic secretory neurons that produce and release gonadotropin-releasing hormone (GnRH) in a pulsatile fashion [5]. Pulsed GnRH release activity depends on excitatory and/or inhibitory transsynaptic and glial inputs from neuronal subsets or glial cells connected to the GnRH neuronal network [5–8]. GnRH peptides stimulate the secretion of gonadotropins, luteinizing hormone (LH), and follicle-stimulating hormone (FSH), from the anterior pituitary gland. These hormones stimulate gonadal maturation and steroid production [9].

The prominent hypothalamic regulatory gene systems involved in the onset of puberty include the leptin [10], γ-aminobutyric acid [4], Lin28 [11], neurohormone B [12, 13], lncRNA [14], and microRNA [15] systems. Lomniczi et al. showed that disrupting the release of pulsatile GnRH in the hypothalamus delayed puberty [16]. Jing et al. showed that the EAP1 gene may be involved in the neuroendocrine control of female puberty in correlation with kisspeptin signaling [17]. These results indicate that the hypothalamus plays an essential role in the onset of puberty.

Proteins are the agents of life and are transformed into their functional form through a series of complex post-translational processing and modification processes. The extensive application of “omics” technology in systems biology has resulted in considerable progress in research on protein function and its complex interactions in response to specific disturbances through global proteome analysis [18]. Proteomics has also been widely used in the study of animal puberty, to elucidate protein expression in uterine fluid [19], ovaries [20], and adipose tissue [21] of pre- and post-pubertal animals. Therefore, quantitative proteomics analysis is a useful tool for understanding puberty onset in animals. The dynamic regulatory events that orchestrate this complex process remain unclear. Many post-translational modifications (PTM), especially protein phosphorylation, which controls biological functions through a variety of mechanisms, are regulated and reversible [22]. Mass spectroscopy-based proteomics could be a valuable tool in the fields of systems biology [23], and extending this approach to proteomics and phosphoproteomics to study the hypothalamus could improve our understanding of the role of hypothalamus in puberty onset.

There have been no studies on quantitative proteomics and protein phosphorylation changes in the prepubertal and pubertal goat hypothalamus to date. We therefore performed a comparative proteomic and protein phosphorylation analysis. Our results could help elucidate the mechanisms of puberty onset, provide a new way for artificial regulation of animal reproduction in animal husbandry. On the other hand, our results could provide a theoretical basis for breeding animal varieties with early puberty and high fecundity, and offer a new perspective for future research into female mammalian reproduction.

## Results

### Proteomic and phosphoproteomic profiles of goat hypothalamus

**Fig. 1:**
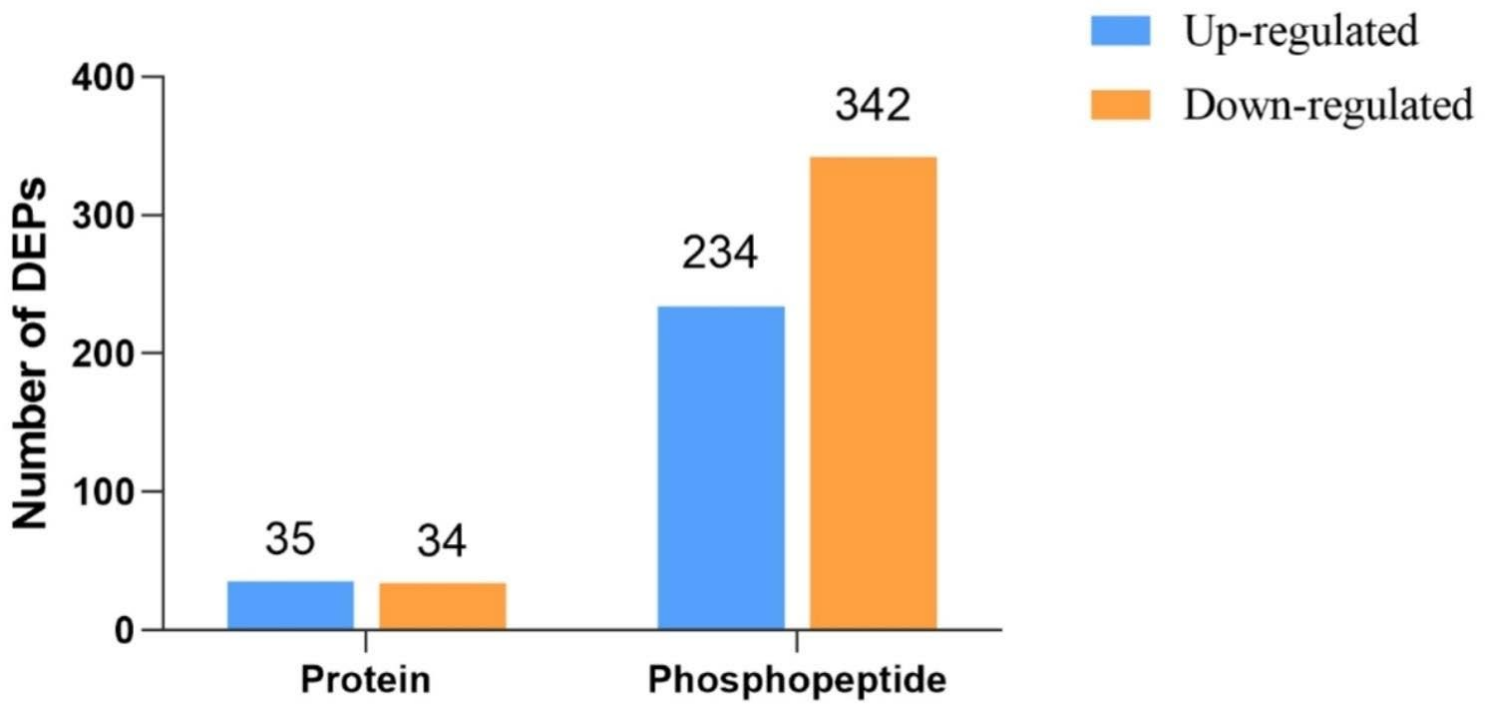
Fold-change distribution of DEPs and DPPs

**Fig. 2:**
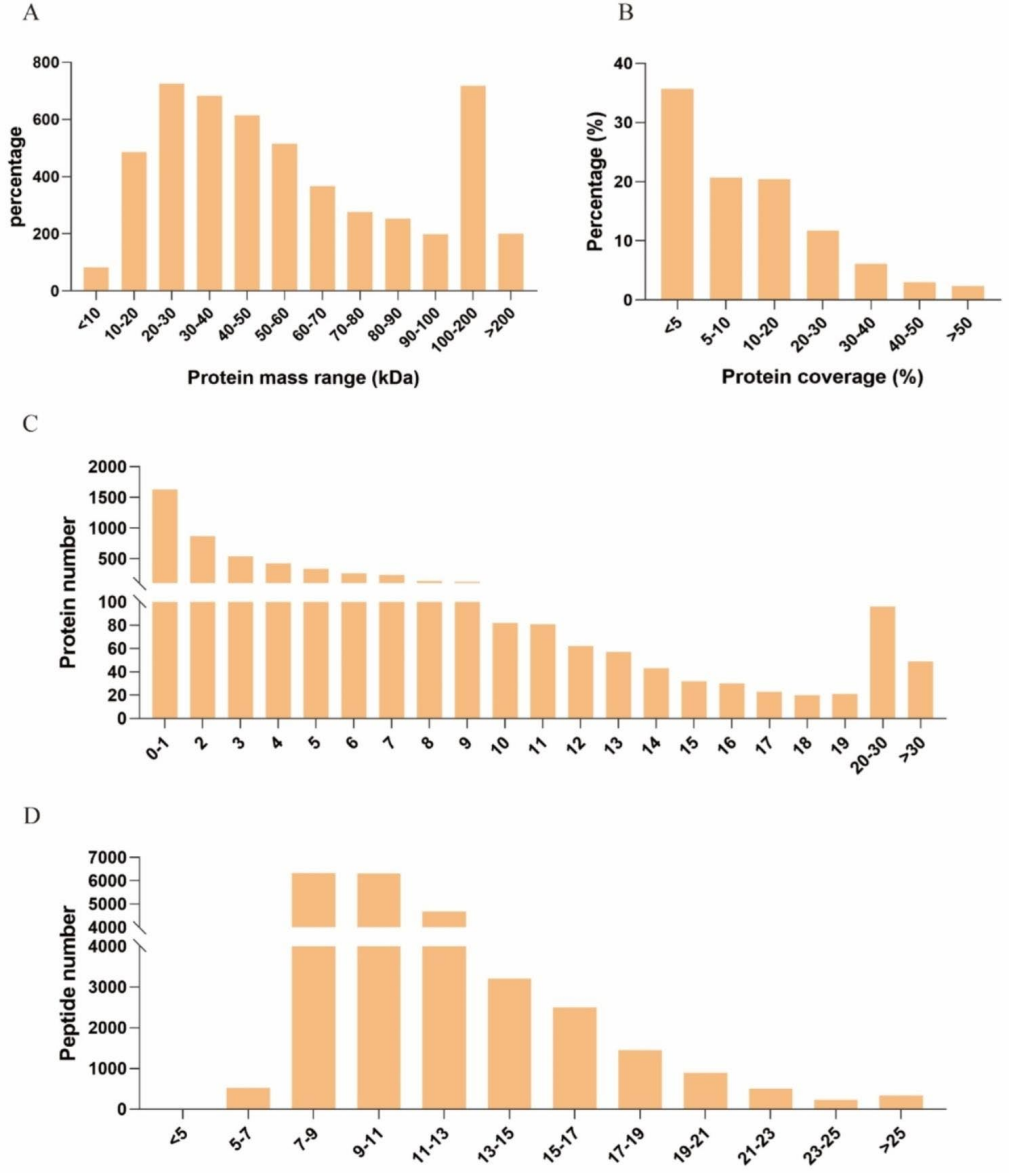
Characteristics of the identified unique peptides in the hypothalamic samples. (**A**) protein mass distribution (**B**) protein coverage (**C**) distribution of unique peptide number (**D**) distribution of peptides based on their length

We compared global protein expression and phosphorylation events in the prepubertal and pubertal goat hypothalamus using 6-Plex TMT-based quantitative proteomics. Three independent biological replicates were tested for each hypothalamus protein sample by TMT labeling. The raw data of proteomic and phosphoproteomic was taken from our previous study on pubertal goats [24, 25]. In total, we identified 24,162 unique peptides (26,928 peptides) from 776,634 spectra corresponding to 5,119 protein species (Table S1 and Table S2). Among these proteins, 5,057 were successfully quantified, and 69 were identified as differentially expressed proteins (DEPs) between the two groups, with a cutoff of 1.2-fold change. 35 of these proteins were significantly upregulated and 34 were significantly downregulated (Fig. 1) [24]. The predicted molecular weights (MW) of the identified proteins varied widely, with a range of 2.3 to 1009.4 kDa and a mean of 67.5.3 kDa (Fig. 2A). The sequence coverage of peptides (Fig. 2B) and the distribution of the peptide number (Fig. 2C) and peptide length (Fig. 2D) were also obtained. More than 68.16% of the identified peptides were detected from at least two unique peptides. In addition, protein sequence coverage with variations > 50%, 40–50%, 30–40%, 20–30%, 10–20%, 5–10%, and under 5% accounted for 2.3%, 3.0%, 6.1%, 11.7%, 20.4%, 20.7%, and 35.7% respectively of the total identified proteins (Fig. 2B).

**Fig. 3:**
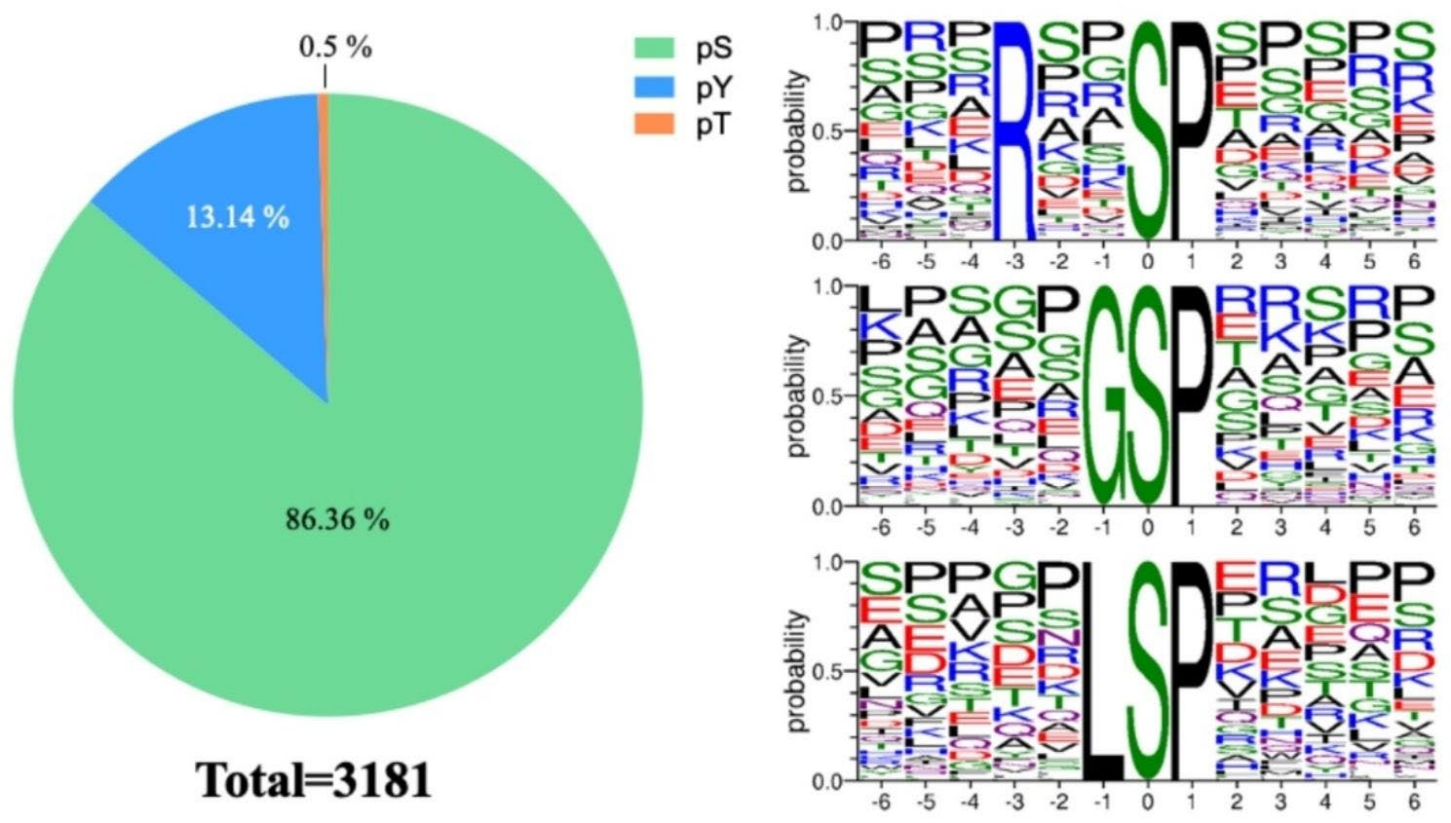
Distribution and phosphorylation motif enrichment of the identified phosphorylation events (**A**) Distribution of phosphorylated serine (pS), threonine (pT), and tyrosine (pY) among the identified phosphorylation events. (**B**) Significantly enriched phosphorylation motifs from all phosphorylation events. The height and color of the residues represent the frequency at the respective positions and their physicochemical properties, respectively

3,040 phosphosites were identified from 3,110 unique phosphopeptides mapping to 1574 proteins of the caprine hypothalamus (Table S3). 385 differentially expressed phosphorylated proteins (DPPs) were detected between the two groups, with a cutoff of 1.2-fold change (Fig. 1). Analysis of the phosphosites of the phosphorylated proteins showed that 883 proteins were phosphorylated at a single site on the protein sequence, and 531 proteins were phosphorylated at two or more sites. MAP1B was identified as a target for 87 phosphorylated sites including 108 phosphopeptides (Table S3) [25]. Consistent with previous reports, the 1,414 assigned sites included 1221 phosphorylated serine, 186 phosphorylated threonine, and 7 phosphorylated tyrosine residues (ratios of 86.36, 13.14, and 0.5% respectively), as shown in Fig. 3.

### Correlated proteins analysis of proteomic and phosphoproteomic profiles

**Fig. 4:**
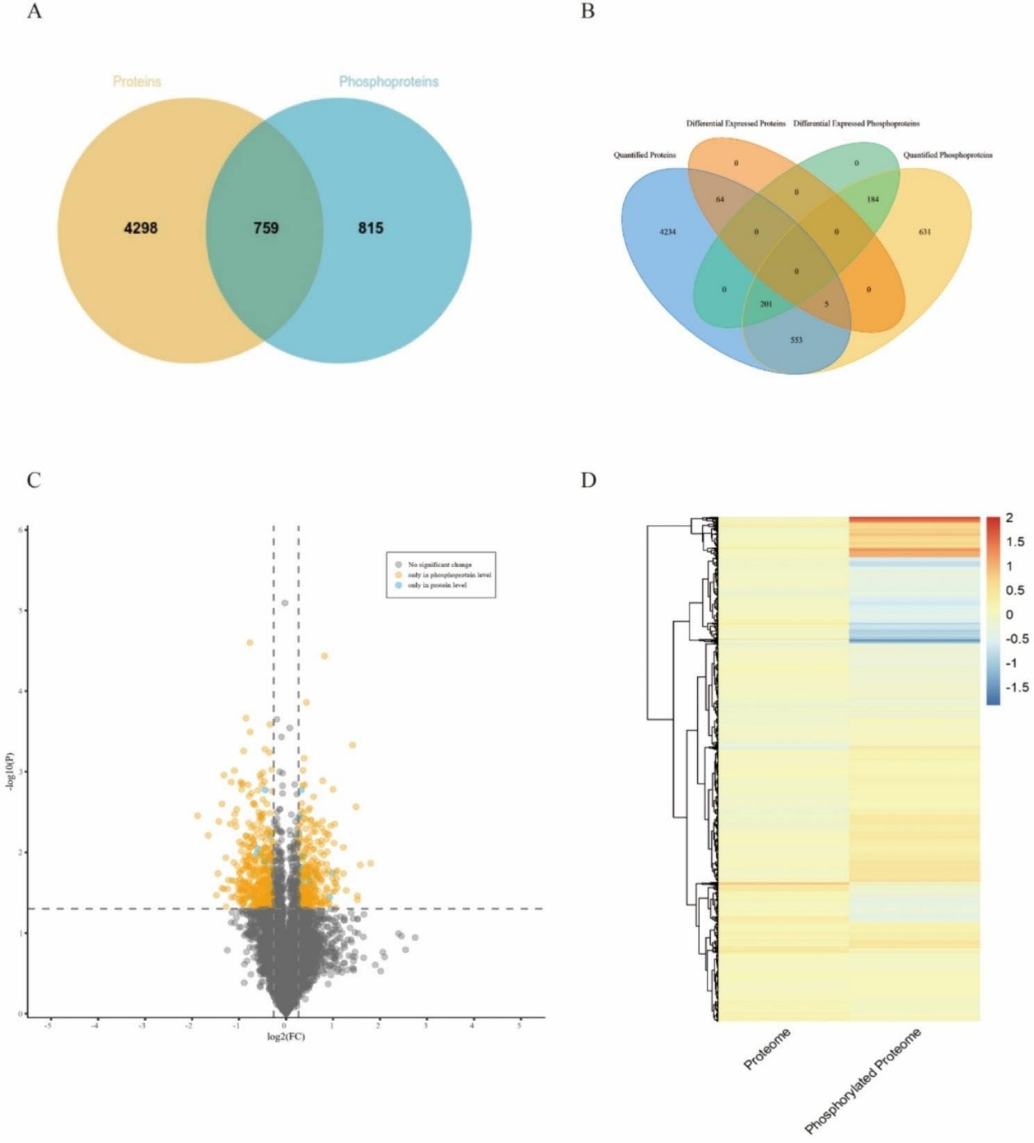
Correlation analysis between proteomic and phosphoprotemic data. (**A**) Venn diagram of the correlation numbers between quantified proteins and phosphoproteins. (**B**) Venn diagram of the number of correlated proteins. (**C**) Volcanic map of differential expression of correlated proteins (differentially expressed only at the protein or phosphoprotein level are shown in the blue and yellow plots, respectively). (**D**) Clustering heat map of expression patterns of associated proteins (red indicates proteins in which expression has increased; blue indicates proteins in which expression has decreased)

All the proteins of the proteomics (5,057) and phosphoproteomics (1574) sets were presented in a Venn diagram (Fig. 4A). We observed 759 correlated proteins (48.2%) which overlapped between the protein and phosphoprotein sets, and about 51.8% of the total phosphoproteins were not detected in the global proteomics analysis (Fig. 4A). Five correlated proteins were differentially expressed only at the protein level (Table 1), 201 were differentially expressed only at the phosphoprotein level, and no proteins were differentially expressed at the protein and phosphoprotein level (Fig. 4B). We used volcano plots to visualize the variation of all correlated proteins in the two omics sets. As shown in Fig. 4C, the blue dot indicates that the correlated proteins differ only in protein expression levels and the yellow dot indicates differences only in phosphoprotein levels. To analyze the distribution characteristics of the correlated proteins at the protein and phosphorylation levels in prepubertal and pubertal goats, as well as the overall differences in protein and phosphorylation expression patterns, correlated proteins were clustered for analysis. The results showed that the expression of proteins containing significantly up-regulated or down-regulated peptides at the onset of puberty (Fig. 4D, Table S4).

**Table 1:** Five correlated proteins were differentially expressed only at the protein level

Protein ID	Protein Name	Gene Name	Protein FC
A0A452EK42	Zinc finger C3HC-type containing 1	ZC3HC1	1.98
A0A452EL94	Dpy-19 like C-mannosyltransferase 1	DPY19L1	1.23
A0A452EDS4	MFS_1_like domain-containing protein	MFSD6	1.23
A0A452EX02	Proprotein convertase subtilisin/kexin type 1 inhibitor	PCSK1N	1.23
A0A452F5Z6	GRIN_C domain-containing protein	GPRIN2	1.21

### Gene ontology (GO) and kyoto encyclopedia of genes and genomes (KEGG) pathway analysis of differentially expressed proteins in the proteome and phosphoproteome

**Fig. 5:**
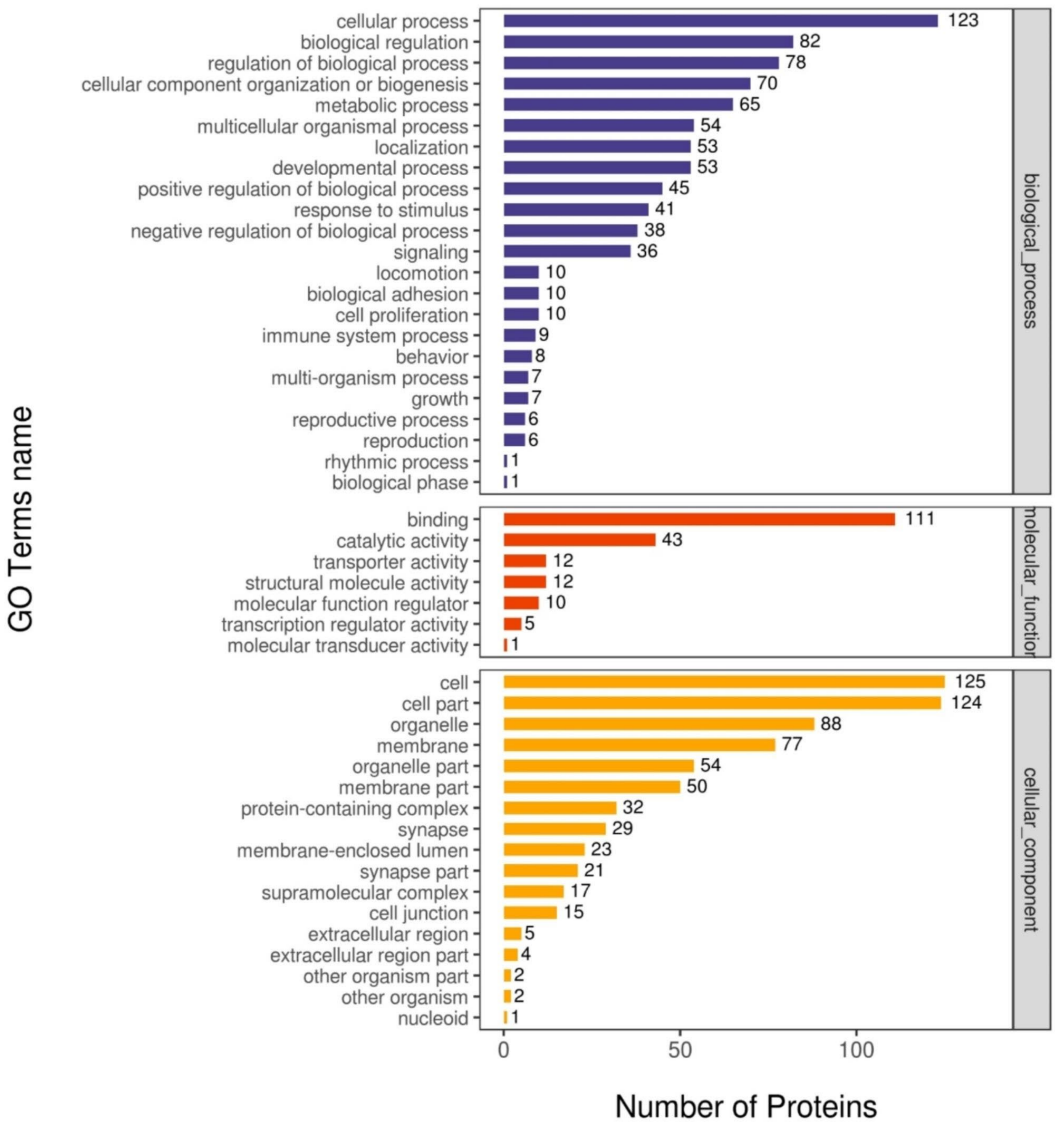
GO enrichment analysis of correlated proteins between prepubertal and pubertal caprine hypothalamus

To understand the biological roles of differential correlated protein phosphorylation in the hypothalamus of prepubertal and pubertal goats, the correlated proteins which were differentially expressed only at the phosphoprotein level and differentially expressed both at protein and phosphoprotein level were annotated by the GO term enrichment and KEGG pathway analysis. First, GO enrichment for correlated proteins with DPPs was conducted, and 47 GO categories were enriched. As shown in Fig. 5, the DPPs were clustered into top GO terms depending on their biological processes, including cellular processes, biological regulation, reproductive process, reproduction, cell proliferation, and growth. The DPPs were classified into top groups based on their cell components; these GO terms included cell junction, protein-containing complex, cell part, synapse, and synapse part. Based on molecular function, the DPPs were classified into 7 groups including transcription regulator activity, molecular function regulator, molecular transducer activity, binding, catalytic activity, structural molecule activity, and transporter activity.

**Fig. 6:**
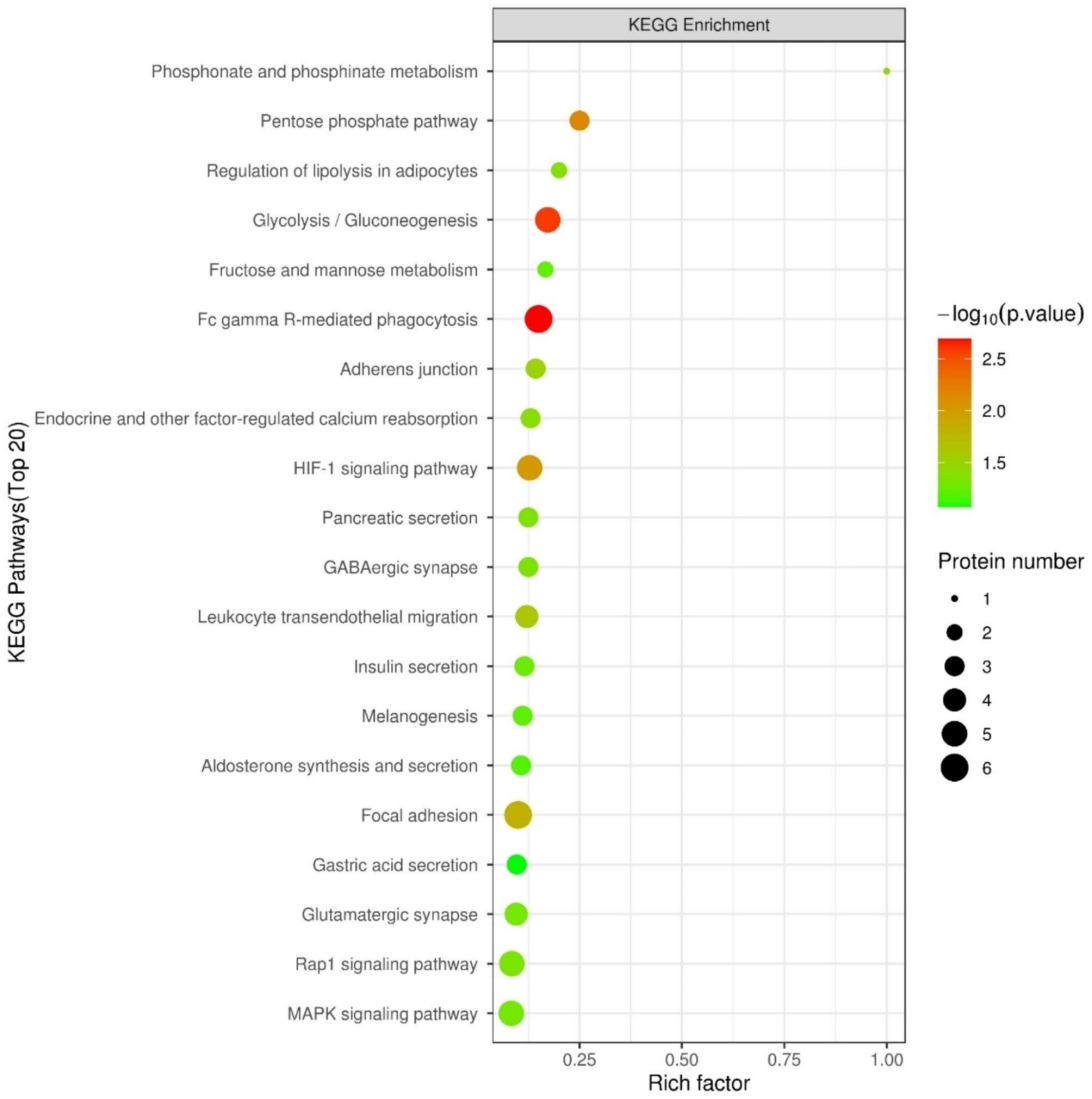
The top 20 KEGG pathways in the correlated proteins between prepubertal and pubertal caprine hypothalamus

The 759 correlated proteins were further analyzed using the KEGG database. We found 20 enriched KEGG pathways (Top 20) at two periods. As illustrated in Fig. 6, many fundamental biological pathways were overrepresented by phosphoproteins identified in this study, including glycolysis/gluconeogenesis, Fc gamma R-mediated phagocytosis, focal adhesion, GABAergic synapse, and Rap1 signaling pathway.

### Phosphoproteome integration in a molecular network

**Fig. 7:**
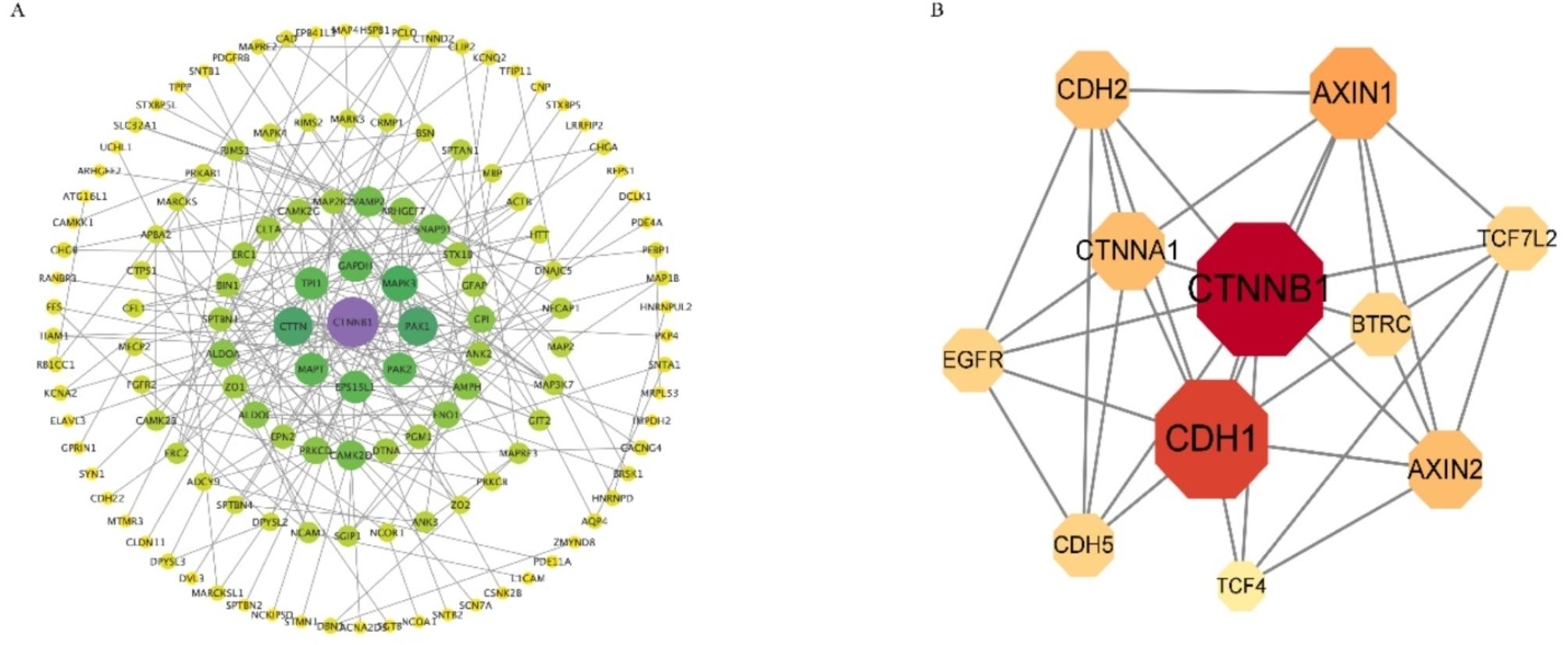
PPI analysis among the correlated proteins (**A**) and CTNNB1 (**B**)

To further investigate the relationships among the identified phosphoproteins, the STRING database was used to identify protein-protein interactions (PPI) and to construct a network of interactions based on a variety of sources including various interaction databases, genetic interactions, text mining, and shared pathway interactions. The DPPs were uploaded for the PPI network construction using STRING (www.string-db.org/), and interactions with at least high confidence were set by default (interaction score > 0.7). As shown in Figs. 7A and 131 proteins were related to 207 paired relationships. CTNNB1 (catenin β-1, also known as β-catenin) occupied the central locations in the PPI network and acted as hubs that interact with other DAPs. CTNNB1, a member of the highly conserved armadillo repeat protein family [26]. CTNNB1 plays a dual role in the cell: as a component of adhesion junction and WNT signaling pathway. Thereby implying that CTNNB1 plays multiple important roles in the regulation of puberty onset. Using the STRING database to further analyze the possible PPI networks of CTNNB1, we identified 10 interrelated proteins (CDH1, CTNNA1, BTRC, AXIN1, CDH2, EGFR, CDH5, TCF4, AXIN2, TCF7L2) in the PPI networks (Fig. 7B).

## Discussion

Timing of puberty has a great influence on the reproductivity of animals. And some evidence has suggested that many proteins in volved in puberty onset. For example, the agonist of NKBR, senktide, stimulated LH release [27] whereas an antagonist of NKBR supressed GnRH/LH pulses in sheep [28]. To our knowledge, this is the first known comprehensive quantitative analysis of the protein and protein phosphorylation differences in the hypothalamus of prepubertal and pubertal goats. The TMT-based proteomics and phosphoproteomics strategy is the powerful technique for the global analysis of signaling networks in defined biological systems [29, 30]. In the field of livestock reproduction, proteomics has been used to determine the molecular basis of hypothalamic function [31], sperm performance [32], and ovarian development [33]. However, an in-depth study of the proteome and phosphoproteome at different developmental stages in the goat hypothalamus has not been reported to date. We performed a global analysis of quantitative proteomics coupled with phosphopeptide-enrichment strategies to study the goat hypothalamus proteome and identify signatures of PTM associated with developmental differences. We identified 69 proteins and 385 phosphoproteins with distinctly altered expression levels in the hypothalamus at the two developmental stages.

In the present study, we identified 5,119 proteins, and quantified 5,057. The total number of identified proteins was similar to the 5,058 proteins identified recently by [31] by using TMT technology, which was substantially more than the 360 proteins identified by [34] using the iTRAQ approach in the pig hypothalamus. The difference in the number of identified proteins is likely because of the difference in species and because of the iTRAQ and TMT approaches, methodological differences used for peptide enrichment detection, and data processing for protein identification and validation. Central AMPK interacts with the puberty-activating gene, Kiss1, to control puberty onset. A vast majority (> 95%) of GnRH neurons co-expressed phosphorylated (p)-AMPK [35] and pubertal subnutrition, which delayed puberty, enhanced hypothalamic pAMPK levels. Activation of brain AMPK in immature female rats substantially deferred puberty [36]. In addition, the expression of p-FOXO1 was markedly downregulated following LKBI overexpression [37]. These findings demonstrate that the onset of puberty is regulated by phosphorylated proteins. PTMs act as signal transducers for adaptations in living organisms because of their reversible and rapid nature, relatively small energy requirement, and effective functional modulation of the target protein [38]. Phosphorylation is the most intensively investigated PTM and is involved in almost every cellular process to orchestrate a variety of cellular signal transduction processes [39]. In the phosphoproteomics, we identified 3,040 phosphopeptides from 1,574 proteins, resulting in the most comprehensive analysis of caprine hypothalamus phosphoproteins to date. According to the results of this study, phosphorylated proteins represent 31.1% of the caprine hypothalamus proteome and are primarily involved in neuron structure or function, structural constituent of cytoskeleton, cAMP signaling, and Rap1 signaling. Data on caprine hypothalamus sites have been very scarce. Therefore, most of the phosphorylation sites identified in the present study are novel protein phosphorylation sites with uncharacterized or unknown functions in the caprine hypothalamus. Thus, the investigation of novel phosphorylation events to elucidate the functions of these hypothalamus phosphoproteins will be of great interest for uncovering the complex regulatory mechanisms involved in caprine hypothalamus function. This could also contribute to elucidating the mechanism of puberty onset in goats.

The correlated proteins identified from the caprine hypothalamus at the two developmental stages by proteomics and phosphoproteomics could help us understand the proteins influencing hypothalamus functionality of genetic factors and provide insights into their functions. In the present study, we identified proprotein convertase subtilisin/kexin type 1 inhibitor (proSAAS), which was differentially expressed only at the protein level, is a neuroendocrine secretory pathway protein. ProSAAS is expressed primarily in the brain and other neuroendocrine tissues (pituitary, adrenal, pancreas) of humans, mice, and rats. In the brain, the mRNA is broadly distributed among neurons. The function of proSAAS includes the inhibition of endogenous prohormone convertases 1, which has catalytic activities in hormone and neuropeptide release [40]. In addition, ProSAAS is cleaved into smaller peptides that may function in cell-cell signaling [41]. Together with these results, our results strongly imply that proSAAS may be involved in the secretion of GnRH.

In this study, GO and KEGG analysis were performed for the correlated proteins. Among them, glycolysis/gluconeogenesis and focal adhesion are important pathways. In the brain, glycolysis/gluconeogenesis mainly occurs in astrocytes, and may provide metabolic fuel in response to glucose deprivation or during increased neuronal transmission [42]. Correlated proteins enriched in glycolysis/gluconeogenesis suggested that more frequent cell communication and more energy may be required during the onset of puberty in goats. Focal adhesion is an important molecular signaling pathway and has a pivotal role in cell proliferation, differentiation, motility, migration, and survival [43]. The formation of a specific structure at the point of contact between the cell and extracellular matrix is called a focal adhesion [44, 45]. In this structure, actin is anchored to transmembrane receptors of the integrin family by a multi-molecular complex, thereby forming a structural connection between membrane receptors and the actin cytoskeleton [46]. Local adhesion of relevant components changes cell shape and gene expression by regulating the actin cytoskeleton [47, 48]. PKCα promotes GT1 neuronal migration by activating focal adhesion complex proteins such as p130^Cas^ and FAK [49]. In addition, focal adhesion is involved in ovulation and fertility in goats [50], and regulate the timing of initiation of primiparity in Nelore bulls [51]. Our results confirmed that focal adhesion has this function in nerve cells and affects GnRH secretion.

CTNNB1, occupies the most important position in the PPI network and involved in focal adhesion, which is an important KEEG pathway in correlated protein analysis. As a component of adherens junctions, it links transmembrane cadherins to the actin cytoskeleton through α-catenin and as an essential component of the WNT signaling pathway, it acts as a transcriptional coactivator in the nucleus [52]. Maudsley et al. used a model HEK293 cell line expressing the GnRH receptor and demonstrated a novel signaling pathway of the GnRH receptor that induces nuclear translocation of the androgen receptor that renders it transcriptionally inactive. This mechanism involves the focal adhesion protein/steroid receptor co-factor, Hic-5 [53]. During brain development, the role of β-catenin in cell adhesions is essential for proper cell migration whereas the WNT signaling pathway regulates cell proliferation and cell fate determination [54–56]. More recently, Elizabeth et al. [42] reported in the journal Developmental Biology that a central and multifaceted role for canonical WNT signaling in regulating growth, patterning, differentiation and nucleogenesis in multiple diencephalic regions. In summary, the above results provide a reliable theoretical basis for CTNNB1 as a new regulated target for the puberty onset of goat.

## Conclusions

In summary, the results of this study reveal multiple differences in the protein level and phosphorylation status of the prepubertal and pubertal hypothalamus of goat, and provide insights into the relationship between goat puberty and reproductive efficiency. Through the parallel and large-scale quantitative analyses of the caprine hypothalamus proteome and phosphoproteome, 759 correlated proteins were identified. In addition, a variety of new molecular mechanisms that be involved in GnRH secretion, neuron proliferation, and puberty onset were identified. To the best of our knowledge, this is the first parallel quantitative proteomics and phosphoproteomics-based study of caprine hypothalamus global proteins at different developmental stages, and these data could improve our understanding of the molecular mechanisms of puberty onset.

## Methods

### Sample preparation and protein extraction

Prepubertal (Pre, n = 3) and pubertal (Pub, n = 3) Anhui White goats (AWGs), an indigenous Chinese breed, were collected from BoDa farm, Hefei, Anhui Province, China. All experimental procedures involving animals were approved by the Animal Care and Use Committee of Anhui Agricultural University (No. AHAU20208025). Sample preparation and protein extraction were performed using previously described procedures [24]. Pre AWGs were 2.5 months of age and weighed 8.1 ± 0.3 kg when tissues were collected, and Pub AWGs were 4.5–5 months of age and weighed 20.16 ± 0.35 kg. Individual natural oestrus was detected in female goats from 4.5 months of age using a male goat, and by observing vaginal physiological changes [57]. Age at puberty onset in female goats was determined when female goats accepted mating, the high level of LH, FSH and E_2_ in serum [58], and vaginal inflammation and mature follicles in the ovaries were observed. After anesthesia by 0.1 ml/10 kg xylazine hydrochloride injection (Lot number 150,804; HuaMu, Jilin, China), goats were sacrificed and the entire hypothalamus was quickly extracted and preserved by snap freezing in liquid nitrogen and stored at − 80◦C until protein extraction.

Whole hypothalamus samples were ground to powder in liquid nitrogen and transferred to a 2 mL centrifuge tube, then lysed in SDT buffer (1 mM DTT, 4% SDS, 100 mM Tris–HCl, pH 7.6). The sample was homogenized on ice by using a MP homogenizer twice (24 × 2.0 ml, 6 m/s, paused for 60 s) and sonicated (80 W for 10 s, paused for 15 s, repeated 10 times). Samples were centrifuged at 14,000 *g* for 10 min at 4℃, and the supernatants were collected. The protein concentration was determined by the BCA assay kit (Bio-Rad) according to the manufacturer’s instructions.

### Trypsin digestion, TMT labeling, and peptide fractionation

Protein digestion was performed according to the previously described filter-aided sample preparation procedure [59]. Briefly, 200 µg of each protein sample was mixed with 200 µl UA buffer (8 M urea, 150 mM Tris–HCl pH8.0) and concentrated for 15 min at 14,000 *g* using 10-kDaultrafiltration centrifuge tubes. The retentates were resuspended in 200 µl UA buffer and concentrated for another 15 min at 14,000 *g* at room temperature (Repeat this step once). 100 µl of 100 mM IAA in UA buffer was added to inhibit reduced cysteine residues, and the samples were incubated for 30 min in darkness and concentrated for 15 min at 14,000 *g*. Subsequently, the filters were washed with 100 µl of UA buffer three times, and 100 µl 25 mM dissolution buffer was added and the samples were spun for 15 min at 14,000 *g* and twice. Finally, the protein suspensions were digested with 40 µl trypsin (4 µg Trypsin in 40 µl dissolution buffer) and placed at 37 ℃ for 16–18 h, and the resulting peptides were collected as a filtrate. The peptide content was estimated by UV light spectral density at 280 nm.

100 µg of peptide from each sample was labelled using TMT 6-plex Isobaric Label Reagent (Thermo Fisher Scientific, Waltham, MA, USA) according to the manufacturer’s instructions. 100 µg of protein per channel were put in new Eppendorf tubes. The TMT labeling reagents were dissolved in 41 µl acetonitrile (ACN, Merck) per vial and added to the samples. The reaction was incubated for 1 h at room temperature and quenched by adding 8 µl of 5% hydroxylamine.

Additionally, peptides were fractionated using strong-cation exchange as described previously [60]. Briefly, strong-cation exchange was performed using a PolySULFOETHYL 4.6 × 100 mm column (4.6 mm × 100 mm, 5 μm, PolyLC Inc, Maryland, U.S.A). Buffer A consisted of 10 mM KH_2_PO_4_ in 25% ACN and pH 3.0, buffer B consisted of 10mM KH_2_PO_4_, 500 mM KCl in 25% ACN and pH 3.0. The following gradient was used: 0–25 min (0% buffer B); 25–32 min (0–10% buffer B); 32–42 min (10–20% buffer B); 42–47 min (20–45% buffer B); 47–52 min (45–100% buffer B); 52–60 min (100% buffer B); 60–120 min (0% buffer B).

### Phosphopeptide enrichment and LC-MS/MS analysis

Phosphorylated peptides were enriched by using TiO_2_ beads (GL Sciences, Japan) as previously described [61]. Peptides were enriched using a High-Select TiO_2_ Phosphopeptide Enrichment Kit (A32993, Thermo Fisher) in accordance with the manufacturer’s procedure. In a nutshell, desalted lyophilized peptides were dissolved in the binding/equilibration buffer included with the kit, and the dissolved peptides were centrifuged to clarify them. Before loading peptides, TiO_2_ spin tips were cleaned three times with wash buffer and once with binding/equilibration buffer. After phosphopeptides had a chance to bind to the TiO_2_ resin, washing steps using binding buffer and wash buffer were performed in order. To avoid dephosphorylation, bound phosphopeptides were then rapidly lyophilized after being eluted with elution solution.

A modest change to the previously described process served as the basis for the LC-MS/MS analysis [62]. The fractionated peptides (after being dissolved in solvent A; 0.1% formic acid) were separated using the the Easy-nLCt 1000 system from Thermo Fisher Scientific (Odense, Denmark), using an Acclaim PepMap100 ((100 μm × 2 cm, nanoViper C18, 3 μm, 100 A; Thermo Fisher Scientific) coupled to an EASY column (10 cm, ID 75 μm, 3 μm, C18-A2; Thermo Fisher Scientific), with a flow rate of 300 nl/min. Using solution B for a sixty minutes elution gradient, the peptides were separated. From 0 to 50 min, the solvent B (0.1% formic acid, 84% acetonitrile) was at 0-35%. And 35-100%, and 100% was from 55 to 60 min, 55 to 60 min, respectively. Peptide signals were analyzed by a Q-exact mass spectrometer with the following parameters (Thermo Fisher Scientific, Bremen, Germany): the resolution of the first mass spectrum was 70,000 at 200 m/z, with the 300–1800 m/z scanning range; secondary mass spectral resolution was 17,500 at 200 m/z; the collision mode was HCD; normalized collision energy 30 eV; and dynamic exclusion duration was 40 s.

### Mass spectrometry data analysis

Proteome Discoverer 2.4 software was used for mass spectrometry data analysis using the Capra hircus UniProt database (35,466 sequences). The retrieval conditions were set as follows: peptide mass tolerance ± 20 ppm; fragment mass tolerance ± 0.1 Da; fixed modification: carbamidomethyl (C); enzyme: trypsin; variable modification: oxidation (M); maximum number of missed trypsin cleavage bits was 2; the false positive rate (FDR) for protein and peptide identification was set at 1%. Furthermore, phosphorylation (S/T/Y) was added as a variable modification in the phosphoproteome analysis.

### Bioinformatics analysis

UniProtKB (http://www.uniprot.org/) database [63] was used to analyze the main functions of the identified proteins. GO annotation was performed using Blast2 GO (https://www.blast2go.com/) [64]. GO annotation was mainly divided into cell components, biological processes, and molecular functions. The regulatory pathways were analyzed using KEGG (https://www.genome.jp/kegg/ ) [65] and KAAS software [66]. String database (https://string-db.org/) was used to obtain the PPI relationships, and the obtained data was imported into Cytoscape 3.2 software to visualize the network.

### Statistical analysis

Analysis of proteomic and phosphoproteomic data was performed by the statistical R package, as well as graphical representations, adopting multiple testing. The significance of data was defined at *p* < .05.

### Electronic supplementary material

Below is the link to the electronic supplementary material.

Supplementary Material 1

Supplementary Material 2

Supplementary Material 3

Supplementary Material 4

## Data Availability

The mass spectrometry proteomics data have been deposited to the ProteomeXchange Consortium via the PRIDE [67]partner repository with the dataset identifier IPX006190000.

## Abbreviations

AWGs: Anhui White goats
DEPs: differentially expressed proteins
DPPs: differentially expressed phosphorylated proteins
FSH: follicle-stimulating hormone
GnRH: gonadotropin-releasing hormone
GO: Gene ontology
HPGA: hypothalamic-pituitary-gonadal axis
KEGG: Kyoto Encyclopedia of Genes and Genomes
LC-MS/MS: Liquid chromatography coupled with Tandem Mass Spectrometry
LH: luteinizing hormone
MW: molecular weights
PPI: protein-protein interactions
Pre: Prepubertal
proSAAS: Proprotein convertase subtilisin/kexin type 1 inhibitor
PTM: post-translational modifications
Pub: pubertal
TiO_2_: titanium dioxide
